# A Large Adrenal Mass in a Patient with Recurrent Acute Heart Failure

**DOI:** 10.1016/j.aed.2025.03.006

**Published:** 2025-04-10

**Authors:** Run Yu

**Affiliations:** Division of Endocrinology, UCLA David Geffen School of Medicine, Los Angeles, California

## Case Presentation

A 66-year-old man was transferred to our institution for a higher level of care. Two days before, he had presented to an outside hospital with shortness of breath for a month. He was found to have acute decompensated heart failure and atrial fibrillation with rapid ventricular response, which quickly deteriorated into cardiogenic shock, respiratory failure, renal failure, and shock liver. He was intubated, required multiple pressors, and was transferred to our institution. He had had acute heart failure, atrial fibrillation, and left ventricular thrombus 5 years before presentation. His heart function had largely recovered until the current illness. He had mild hypertension and diabetes but no history of coronary artery disease. His family history was reportedly “unremarkable.” At our institution, he appeared to be in critical distress but was not Cushingoid. Veno-arterial extracorporeal membrane oxygenation and continuous renal replacement therapy were promptly initiated. Afterward, results of a computed tomography (CT) scan of the chest performed for ruling out pulmonary embolism at the outside hospital arrived and reportedly showed liver and left retroperitoneal masses. A CT scan of the chest with contrast performed at our institution showed cardiomegaly, small loculated pericardial fluid, and bilateral pleural effusion (Fig. *A*). A CT scan of the abdomen and pelvis with contrast showed a heterogeneously enhancing left adrenal soft tissue mass measuring 8.2 × 6.7 cm (Fig. *B*, ∗) and multiple scattered cysts in the liver (Fig. *B*, #). A CT scan of the adrenal glands showed the left adrenal mass exhibited a precontrast Hounsfield unit >40 and heterogeneous enhancement (Fig. *C*, *D*). The right adrenal gland was normal.

**Fig:**
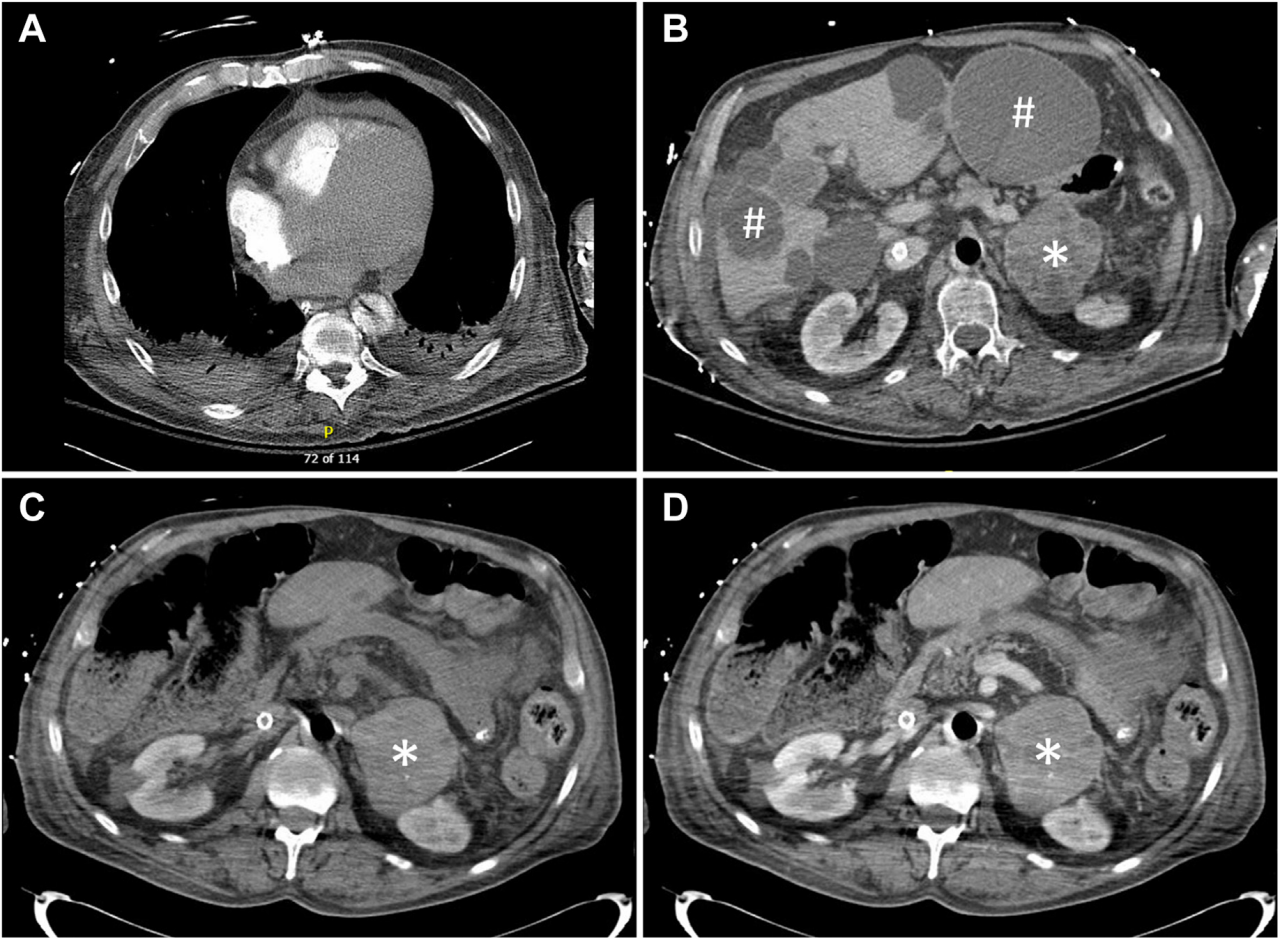

## What is the diagnosis?

### Answer

Left adrenal pheochromocytoma causing recurrent cardiomyopathy. In a patient with unexplained cardiomyopathy, a large adrenal or retroperitoneal mass is pheochromocytoma or functional paraganglioma until proved otherwise.^1^ His plasma metanephrine was 13.90 nmol/L (normal <0.49), normetanephrine 25.40 nmol/L (normal <0.89), dehydroepiandrosterone sulfate 1140 ng/mL (normal 500-4000), and aldosterone 7.8 ng/dL (normal <16) while receiving an epinephrine infusion at 0.05 μg/kg/min. Although critical illness and pressors can raise metanephrine levels a few-fold, his extremely high metanephrine levels confirmed the pheochromocytoma diagnosis.^2^ In retrospect, his cardiac presentations 5 years before were also certainly due to pheochromocytoma-induced cardiomyopathy, which can be self-limiting initially but become catastrophic later.^1^ Although pheochromocytoma can metastasize to the liver, cystic pheochromocytoma metastatic lesions are very rare and should exhibit peripheral enhancement, which was absent in this patient.^2^^,^^3^ The multiple liver cysts were thus more likely due to polycystic liver disease. Despite aggressive cardiovascular support with veno-arterial extracorporeal membrane oxygenation and intra-aortic balloon pump and cautious alpha blockade with doxazosin 1 mg (a higher dose could not be tolerated due to hypotension), the patient died of multiorgan failure 3 weeks after transfer to our institution. This case thus highlights that a large adrenal mass in otherwise unexplained cardiomyopathy is pheochromocytoma by default.

## Disclosure

The author has no conflicts of interest to disclose.
